# Supplementary material for: Structural motifs for subtype-specific pH-sensitive gating of vertebrate otopetrin proton channels
Source: eLife. 2022 Aug 3;11:e77946. doi: 10.7554/eLife.77946 (PMC9348849; doi:10.7554/eLife.77946)
Supplement: Figure 6—source data 2. — P values and sample size are indicated. [file elife-77946-fig6-data2.docx]

**Figure 6 - Supplementary Table 1**. Statistical tests comparing chimeric channels with wildtype channels with Mann-Whitney U test. P values and sample size are indicated. —

* *NA* indicates no measurable currents.

** The numbers are labeled in bold if P < 0.05.

|  | **OTOP2** | | **OTOP3** | |
| --- | --- | --- | --- | --- |
|  | pH 10 | pH 6 | pH 10 | pH 6 |
| L1-2 | 0.063492  (N = 5) | 0.730159  (N = 5) | NA | NA |
| L3-4 | NA | NA | **0.015873**  **(N = 4)** | **0.035714**  **(N = 3)** |
| L5-6 | **0.028571**  **(N = 4)** | **0.028571**  **(N = 4)** | 0.063492  (N = 4) | **0.015873**  **(N = 4)** |
|  |  |  |  |  |
| L7-8 | 0.685714  (N = 4) | 0.999999  (N = 4) | 0.690476  (N = 5) | **0.015873**  **(N = 5)** |
| L9-10 | NA | NA | 0.841270  (N = 5) | **0.007937**  **(N = 5)** |
| L11-12 | 0.171429  (N = 6) | 0.761905  (N = 6) | 0.555556  (N = 4) | **0.004329**  **(N = 6)** |
